# Comment on “Respiratory mechanics and gas exchanges in the early course of COVID-19 ARDS: a hypothesis-generating study”

**DOI:** 10.1186/s13613-020-00765-6

**Published:** 2020-10-23

**Authors:** Elisa Damiani, Andrea Carsetti, Erika Casarotta, Roberta Domizi, Claudia Scorcella, Erica Adrario, Abele Donati

**Affiliations:** Anesthesia and Intensive Care Unit, Department of Biomedical Sciences and Public Health, Università Politecnica delle Marche, via Tronto 10/a, 60126 Ancona, Italy

Dear Editor,

We have read with great interest the article by Diehl et al. on the evaluation of respiratory mechanics and gas exchanges in patients with acute respiratory distress syndrome (ARDS) due to COVID-19 that was recently published in the *Annals of Intensive Care* [1]. In 22 patients with moderate-to-severe ARDS, the authors observed high physiological dead space (*V*_D_/*V*_T_) and ventilatory ratio (VR). Several hypotheses are made to explain the pathogenesis of increased *V*_D_/*V*_T_, namely pulmonary embolism, alveolar overdistension, increased instrumental dead space, and diffuse microcirculatory dysfunction [1]. Even if alveolar overdistension due to high PEEP-protective ventilation, with regional compression of alveolar vessels, is likely to exert a major impact on lung mechanics in ARDS, the hypothesis of a contributing role of microvascular derangement is particularly captivating.

Lung injury in ARDS (and COVID-19 pneumonia) seems mainly driven by a dysregulation of the renin–angiotensin system, leading to increased vascular permeability, inflammation, pneumocyte apoptosis, and fibrosis [2]. Pulmonary microvascular injury, with leaky blood vessels, interstitial oedema, microthrombosis, and heterogeneous perfusion, may be the first responsible for ventilation/perfusion mismatch and increased dead space in ARDS. In 42 patients with early moderate or severe ARDS, Ospina-Tascon et al. showed an inverse correlation between *V*_D_/*V*_T_ and sublingual microcirculatory blood flow distribution [3]. In their study, Diehl et al. reported an elevation of markers of endothelial damage and thrombosis (i.e., circulating endothelial cells and D-dimers); however, they did not show any statistical correlation with variables of respiratory mechanics and/or gas exchange [1].

In a recent report, we described the sublingual microcirculation of mechanically ventilated patients with severe SARS-COV-2 pneumonia and showed an inverse correlation between perfused vessel density (PVD) and D-dimers [4]. This relationship was confirmed in SARS-COV-2 patients on veno-venous extracorporeal membrane oxygenation [5]. Unfortunately, we could not evaluate the relationship with physiological dead space, because *V*_D_/*V*_T_ data were not available for our cohort. We calculated the VR for 6 patients who were not receiving extracorporeal membrane oxygenation (median VR = 1.9 [1.4–2.5]); however, we could not find any significant correlation with D-dimers (Spearman’s rho = − 0.1, *p* = 0.950) or microcirculatory variables (Spearman’s rho for PVD = 0.232, *p* = 0.650). The extremely low sample size significantly limits these analyses.

Taken together, all these data would suggest a connection between microvascular dysfunction, coagulopathy, and increased physiological dead space in the genesis of respiratory failure in COVID-19 pneumonia. Nonetheless, the cause–effect relationship remains to be proven. An altered sublingual microcirculation could just be an epiphenomenon of a hemodynamic compromise in patients with worse respiratory mechanics: in our cohort, sublingual microvascular perfusion tended to decrease with increasing driving pressures [4]. Further investigations are imperative to gain a more comprehensive understanding of the pathophysiology of COVID-19 and select the best treatment strategy. Appropriately designed clinical and laboratory-controlled studies are needed to prove any causal relationship between microvascular derangements and increased dead space ventilation.

Finally, a consideration must be made regarding the article by Diehl et al. [1]: the authors used side-stream capnography, which may be inaccurate for calculations of *V*_D_/*V*_T_ as compared to main-stream capnography. The transport delay of the gas in the sampling tube with axial mixing of the gas residing in the tube, together with the variable sampling flow rate resulting from the alternating positive airway pressure during mechanical ventilation, leads to underestimation and distortion of the capnogram, with consequent inaccurate *V*_D_/*V*_T_ calculations.

## Data Availability

Not applicable.

## Abbreviations

ARDS: Acute respiratory distress syndrome
COVID-19: Severe coronavirus disease 2019
*V*_D_/*V*_T_: Physiological dead space
VR: Ventilatory ratio
SARS-COV-2: Severe Acute Respiratory Syndrome Coronavirus 2
PVD: Perfused vessel density
